# Supplementary figures and images for: miRNA expression in advanced Algerian breast cancer tissues
Source: PLoS One. 2020 Feb 10;15(2):e0227928. doi: 10.1371/journal.pone.0227928 (PMC7010257; doi:10.1371/journal.pone.0227928)

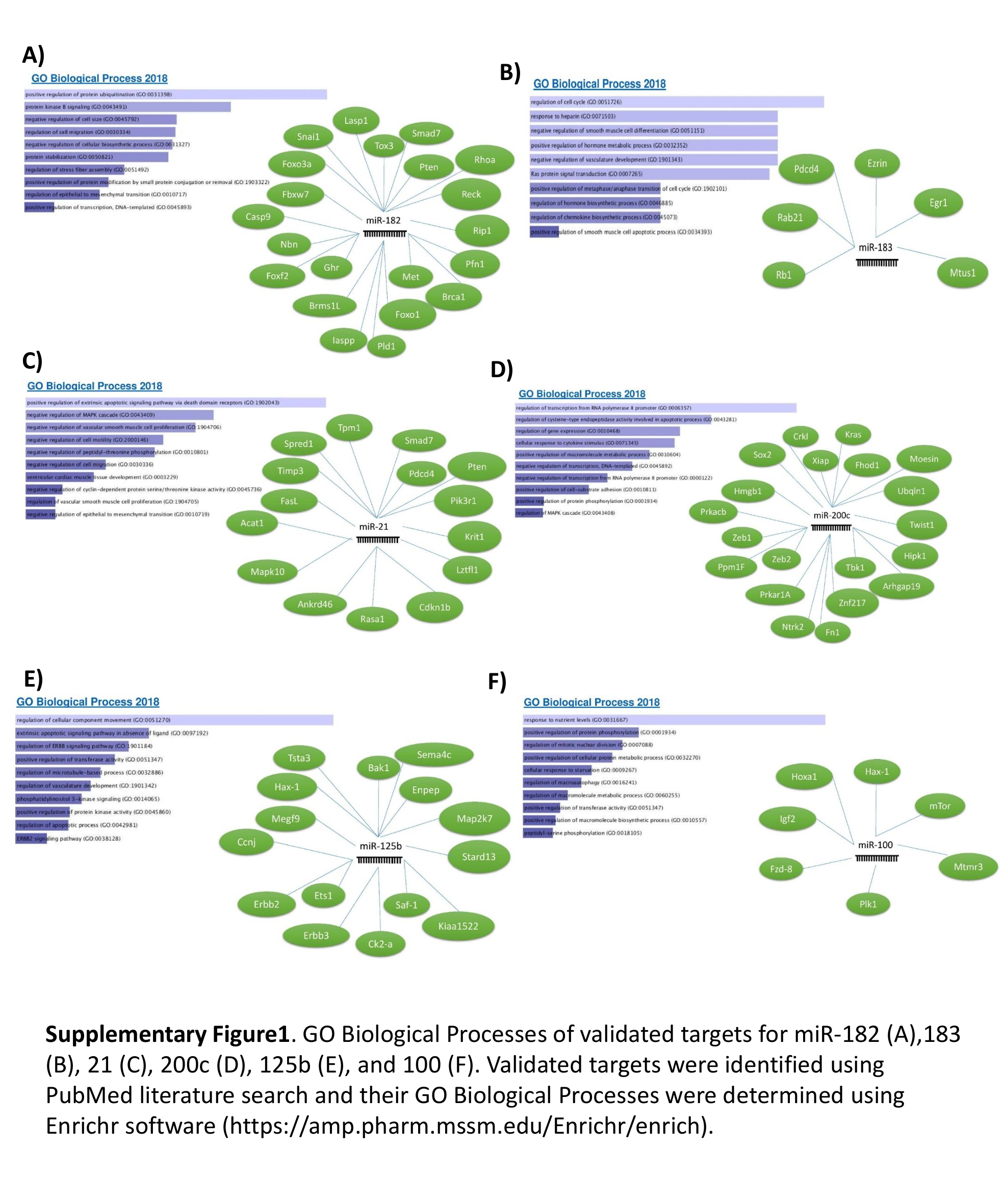

Supplement: S1 Fig — Validated targets were identified using PubMed literature search and their GO Biological Processes were determined using Enrich software (https://amp.pharm.mssm.edu/Enrich/enrich). (TIF) [file pone.0227928.s001.tif]
